# Mechanistic insight into radical *S*-adenosylmethionine enzymes in lipid modification

**DOI:** 10.1039/d6np00037a

**Published:** 2026-08-11

**Authors:** Tamra Blue-Lahom, Jiayuan Cui, Cody T. Lloyd, Squire J. Booker

**Affiliations:** a Department of Chemistry, School of Arts and Sciences, University of Pennsylvania Philadelphia PA USA booker1@sas.upenn.edu; b Department of Biochemistry & Molecular Biology, The Pennsylvania State University, University Park PA USA; c Department of Biochemistry and Biophysics, Perelman School of Medicine, University of Pennsylvania Philadelphia PA USA; d Department of Chemistry, The Pennsylvania State University, University Park PA USA; e Howard Hughes Medical Institute Chevy Chase MD USA

## Abstract

Covering: up to 2025

Lipids are structurally diverse biomolecules that play a critical role in the homeostasis of organisms across all domains of life. While biogenesis of lipids varies by organism, the strategies employed to modify or functionalize inert lipid hydrocarbon chains often require the power of radical-based chemistry. Recently, an increasing number of lipid-modifying radical *S*-adenosylmethionine (RS) enzymes have been identified. RS enzymes are well known to mediate >100 reactions. In the context of lipids, these radical-mediated reactions proceed through Csp^2^ and Csp^3^ centers, affording C–C bond formations, methylations, cyclizations, and/or ring contractions. This review highlights our understanding of the function and mechanism of lipid-modifying RS enzymes. Mechanistic considerations of lipid modification by RS enzymes reveal the significance of cofactors (particularly B12) and auxiliary metal centers in substrate activation and intermediate stabilization. Understanding the function and mechanism strategies of these enzymes is crucial not only to advancing our understanding of RS enzymes but also to inform paleoenvironmental reconstructions and the emerging field of lipid-based drug delivery.

## Abstract 2

This review is a comprehensive overview of radical *S*-adenosylmethionine enzymes involved in lipid modification. The unique mechanistic strategies of these enzymes, the native function of lipids, and the current advances in the field are highlighted.

## Graphical abstract

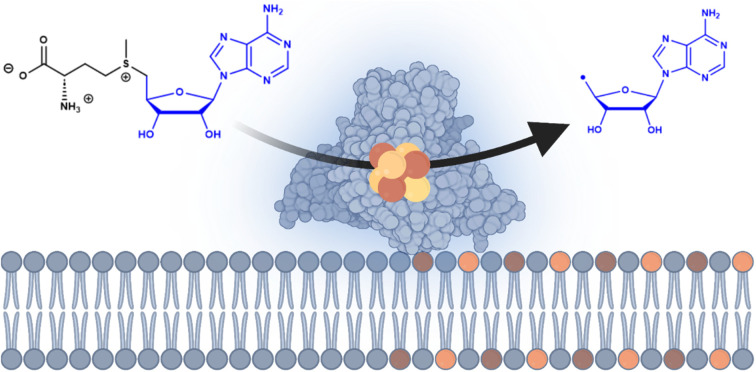

## 1. Introduction

**Fig. 1:**
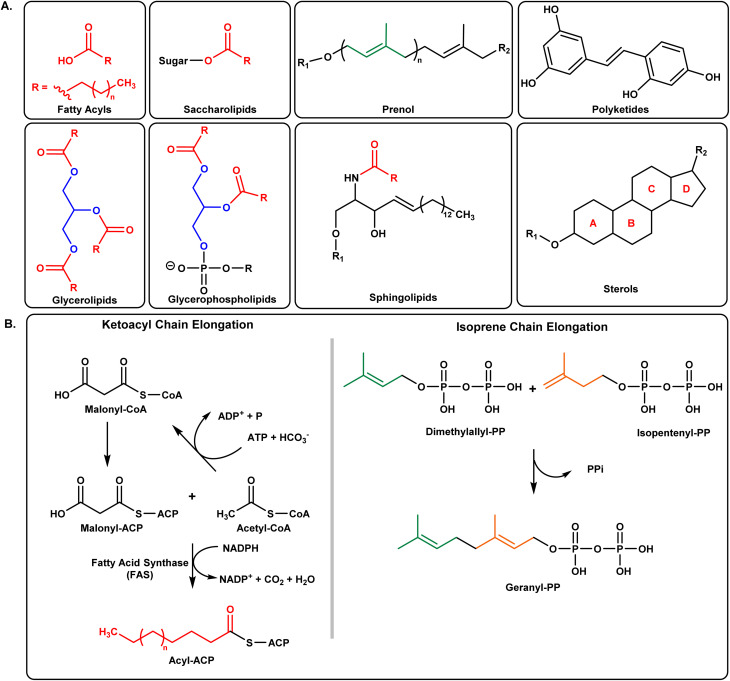
(A) Eight general lipid categories according to the LIPID MAPS classification system: fatty acyls or fatty acids (FA), saccharolipids, prenol lipids, polyketides, glycerolipids, glycerophospholipids, sphingolipids, and sterol lipids. (B) Chain elongation mechanisms from ketoacyl and isoprene building blocks.

Lipids are structurally and functionally diverse hydrophobic molecules that play essential roles in biological systems.^1^ While they are best known as the primary constituents of cellular membranes, lipids also function in energy storage, signaling, molecular transport, antimicrobial defense, enzyme activation, and numerous other processes.^2^ The Lipid Metabolites and Pathways Strategy (LIPID MAPS) categorizes lipids into eight major classes: fatty acyls (fatty acids), glycerolipids, glycerophospholipids, sphingolipids, sterol lipids, prenol lipids, saccharolipids, and polyketides (Fig. 1A).^3,4^ Fatty acids (FAs) serve as the foundational building blocks of glycerolipids, glycerophospholipids, sphingolipids, and saccharolipids, all of which are involved in membrane structure or energy storage.^5,6^ In addition to their biological functions, lipids possess chemical properties that make them valuable in biomedical applications.^7^ They are widely used as excipients in pharmaceutical formulations, including nanoemulsions, liposomes, and solid lipid nanoparticles, to improve the solubility and bioavailability of poorly water-soluble drugs.^8^ Despite their significance in biology and medicine, lipids are relatively underexplored compared to proteins, nucleic acids, and carbohydrates.^9^

Lipids also play an important role in paleoenvironmental reconstruction as molecular fossils and biomarkers for reconstructing past environmental conditions.^10,11^ Just as an archaeologist uses ancient artifacts to reconstruct features of past civilizations, preserved organic molecules—or “molecular fossils”—from ancient organisms can be used to reconstruct a timeline of environmental pH, temperature, and salinity.^12,13^ Lipids are also used to track changes in biodiversity and the appearance of major organismal groups.^12^ As biomarkers, these molecular fossils must be stable and resistant to extreme environmental variations—an innate feature of lipid hydrocarbon chains. Understanding how lipids are biosynthesized and the environmental conditions that influence lipid diversity is, therefore, essential for interpreting their physiological functions and their utility as ecological proxies.

### 1.1 Lipid structure and biosynthesis

All lipids are synthesized from two types of chemical precursors: ketoacyl building blocks or isoprene building blocks (Fig. 1B). The fatty acyl chains of ketoacyl-based lipids are synthesized from acetyl coenzyme A (CoA) and malonyl-CoA. In a six-step enzymatic cascade, the alkyl groups of these molecules are transferred to a separate acyl carrier protein (ACP) (type II FA biosynthesis) or ACP module (type I FA biosynthesis) and combined to generate a saturated chain consisting of four carbons. Each subsequent round of elongation by an additional molecule of malonyl-CoA extends the fatty acyl chain by two carbons. The simplest ketoacyl-based lipids are FAs, comprising a single fatty acid moiety. After elongation of the hydrocarbon chain, the fatty acid is liberated from the ACP *via* hydrolysis to yield a free acid with a terminal carboxyl group or transferred to a molecule of CoA (Fig. 1B). However, certain functionalizations of the carboxyl group can generate new lipid categories. Saccharolipids are formed when one or more FA chains are directly linked to a monosaccharide backbone. Similarly, two FA chains can be linked to the amine and carboxyl groups of a serine amino acid, resulting in a sphingolipid. Sphingolipid subclasses include sphingomyelin (phosphate head group) and glycolipids (carbohydrate head group).

In contrast to liberating free FA for their further modification, the direct transesterification of an acyl-ACP to a glycerol backbone is the first committed step in synthesizing glycerolipids and glycerophospholipids. Glycerolipids are composed of a glycerol backbone esterified with one to three fatty acyl chains. The presence of a glycerol backbone is the defining structural characteristic of the glycerolipids category. However, if the glycerol backbone contains a phosphate group, which results from appending the fatty acyl chains to a molecule of glycerol-3-phosphate, the resulting lipid is defined as a glycerophospholipid. The glycerophospholipid contains a glycerol backbone with one to two ester-linked fatty acyl chains and a terminal phosphate.^4^ Unlike the other categories of ketoacyl-based lipids, the polyketides are cyclized lipids that result from the polymerization of various acyl-CoA building blocks, such as malonyl-CoA and methylmalonyl-CoA.

The remaining two lipid categories, sterols and prenols, are derived from the five-carbon isoprenes dimethylallyl pyrophosphate (DMAPP) and isopentenyl pyrophosphate (IPP).^14,15^ DMAPP and IPP are synthesized from acetyl-CoA *via* mevalonate or non-mevalonate pathways. A single enzyme, geranylgeranyl pyrophosphate (GGPP) synthase, condenses IPP and DMAPP into the ten-carbon chain observed in geranyl pyrophosphate (GPP). Subsequent rounds of elongation add one or two molecules of DMAPP to the chain, generating the farnesyl pyrophosphate (FPP, C15) and GGPP (C20) chains, respectively. Prenol lipids comprise one or more five-carbon isoprene units, which can be linked to glycerol, glycerophosphate, and sugar backbones, generating the glycerol-, glycerophosphate-, and saccharide lipid ketoacyl equivalents. Yet, LIPID MAPS categorizes these lipids under the prenol lipid differentiation (Fig. 1A). The other category of isoprenoid-based lipids is sterols, which are steroid-derived lipids composed of a steroid nucleus, a perhydrophenanthrene molecule linked to a four-membered ring.^16,17^ After forming two molecules of FPP, squalene synthase couples them to generate squalene. The resulting thirty-carbon isoprenoid chain is cyclized by squalene cyclase to generate the four-ringed backbone of the steroid nucleus.

The eight lipid categories are further differentiated by their polar or non-polar head groups, linked chemical moieties (R), chain length, degree of unsaturation, and level of chain branching or cyclization. The biosynthesis of basic lipid structures relies solely on polar chemistry, often involving Aldol and Claisen-type reactions. However, further elaboration or maturation of the inert lipid hydrocarbon chain often requires radical-based chemistry. Nature uses several strategies to initiate radical-based reactions, such as that employed by the radical *S*-adenosylmethionine (SAM) enzyme superfamily. Herein, we explore our mechanistic understanding and analysis of the reactions catalyzed by radical SAM (RS) enzymes involved in lipid modification.

### 1.2 The RS superfamily

**Fig. 2:**
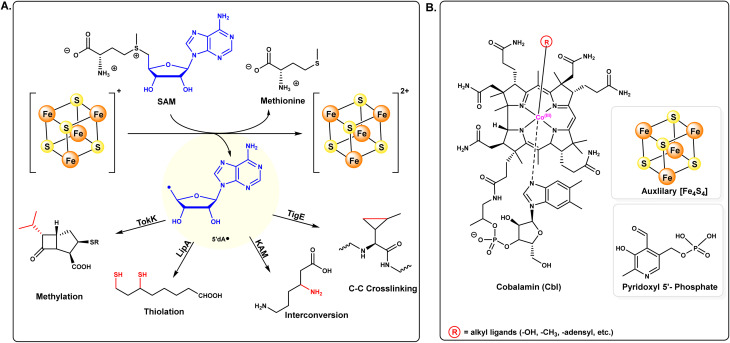
(A) Canonical reductive cleavage of SAM by RS enzymes to form 5′dA· (key examples methylation (TokK),^32^ thiolation (LipA),^32^ amino group migration (KAM),^32^ and C–C crosslinking (TigE);^32^ RS enzyme modifications in red). (B) Common RS cofactors: cobalamin, auxiliary [Fe_4_S_4_] clusters, pyridoxal 5′-phosphate (PLP).

There are more than 450 000 members of the RS superfamily annotated in the InterPro consortium.^18^ According to https://radicalSAM.org, the RS superfamily consists of over 42 distinct subgroups.^19^ RS enzymes are typically identified by a conserved Cx_3_Cx_2_C motif containing the cysteine ligands that bind a requisite [Fe_4_S_4_] cluster. This iron–sulfur (Fe/S) cluster, termed the [Fe_4_S_4_]_RS_ cluster, is housed within a full or partial triose-phosphate isomerase (TIM) barrel.^19,20^ Three of the cysteines bind to three of the four Fe ions of the cluster, leaving a unique Fe to which *S*-adenosylmethionine (SAM) binds through its amino and carboxylate groups. In the reduced state, the [Fe_4_S_4_]_RS_^+1^ cluster induces a homolytic cleavage of the S–C5′ bond of SAM to form a transient 5′-deoxyadenosyl 5′-radical (5′-dA·) and methionine (Met). The highly reactive 5′-dA· typically initiates catalysis by abstracting a hydrogen atom (H·) regio- and stereo-selectively from a nearby, often unreactive, atom on the substrate (Fig. 2A). This H· abstraction generates a substrate radical that often reacts with additional substrates or cofactors en route to product formation. RS enzymes catalyze >100 unique reaction types, including oxidative decarboxylations,^21^ sulfur additions,^23,24^ thioether bond formations,^25,26^ methylations,^27,28^ methylthiolations,^29^ C(sp^3^)–C(sp^3^) bond formation,^30^ and cyclization, among others^31^ (Fig. 2A).

The broad scope of RS reactions is enhanced by the deployment of cofactors such as cobalamin (Cbl), pyridoxal 5′-phosphate (PLP), and auxiliary Fe/S clusters (Fig. 2B).^24,33^ Cbl-dependent RS enzymes constitute one of the largest subgroups within the superfamily. While most are annotated as methylases,^28,34^ Cbl-dependent RS enzymes are involved in ring contractions,^35–37^ oxidation reactions,^38^ and cyclizations.^31,38,39^ By contrast, PLP is used to stabilize substrate radicals to allow for functional group migrations, such as in the RS enzyme lysine 2,3-aminomutase (LAM or KAM).^40,41^ KAM catalyzes the interconversion between (*S*)-lysine and (*S*)-β-lysine, with PLP acting as a scaffold that binds (*S*)-lysine while stabilizing the substrate radical intermediates.^42,43^ The auxiliary Fe/S clusters of RS enzymes play various roles, from acting as sulfur sources,^44^ electron transfer,^22,32,45–47^ stabilizers of high-energy intermediates,^30^ and substrate binding.^48^ The high oxidizing power of the 5′-dA· permits RS enzymes to catalyze an array of kinetically challenging reactions, such as the modification of chemically inert hydrocarbon chains. In the past four decades, a clearer picture has emerged regarding the importance of RS enzymes in lipid biogenesis.

## 2. Glycerol tetraether lipids

Archaea can thrive under extreme environmental conditions such as elevated temperatures, salinities, and low or high pH values, where eukaryotes and bacteria often struggle to survive.^49^ Several unique lipid membrane features confer archaea with enhanced fitness in such environments. The majority of membrane lipids comprise a glycerol-1-phosphate backbone linked by an ether bond to isoprenoid tails known as phytanyl chains.^50^ Unlike the ester bonds of eukaryotic and bacterial membrane lipids, the ether bonds of archaeal lipids are stable to high temperatures and acidic conditions, preventing membrane lipid hydrolysis. Instead of a lipid bilayer, some archaea are known to exhibit a monolayer consisting of membrane-spanning lipids known as glycerol dialkyl (or dibiphytanyl) glycerol tetraether (GDGT). GDGT lipids promote adaptation to harsh environmental conditions such as high temperature and acidity. Studies indicate that archaea augment the ratio of GDGT to diether lipids under high growth temperatures to promote membrane thermal stability.^51^ Although primarily in archaeal organisms, GDGT-like lipids, also known as branched-GDGTs (brGDGTs), have also been identified in select hyperthermophilic bacteria. Like their archaeal homologs, these bacterial GDGT-like lipids are membrane-spanning and contain ether bonds rather than the expected ester bonds.^52^

**Fig. 3:**
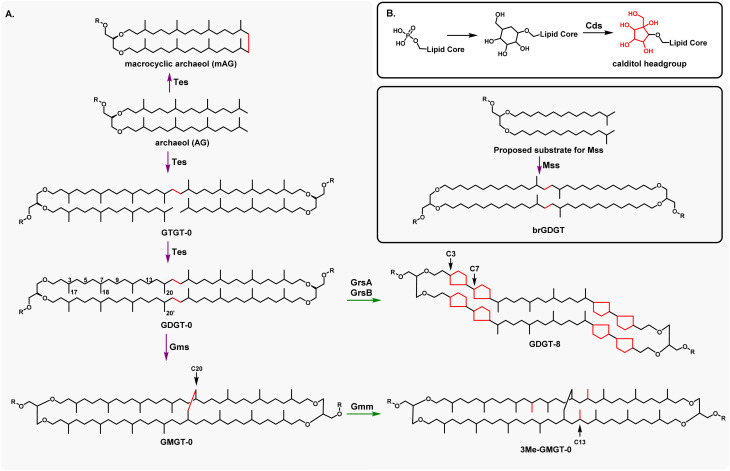
RS enzymes involved in membrane lipid core (A) and headgroup (B) biosynthesis or modification: mAG: macrocyclic archaeol; AG: archaeol; GTGT: glycerol trialkyl glycerol tetraether; GDGT: glycerol dialkyl glycerol tetraether, numbers assigned represent the cyclopentyl moiety insertions; GMGT: glycerol monoalkyl glycerol tetraether; brGDGT. RS enzyme-catalyzed modifications are labeled in red. Purple arrows indicate the auxiliary [Fe_4_S_4_] cluster-dependent mechanism, while green arrows indicate cobalamin. (R = phosphate head group).

GDGTs are prenol lipids, comprising two branched isoprenoid fatty alkyl chains connected by four ether bonds to two glycerol backbones, often with a phospho- (PO_4_-R) and/or glyco-head group. GDGTs can include additional modifications, such as up to eight cyclopentane rings and carbon–carbon bond crosslinks between the two biphytanyl chains (Fig. 3). These lipids are commonly annotated as GDGT-*n*, where *n* corresponds to the number of cyclopentane moieties. Studies by Boyd *et al.* showed that the number of cyclopentane rings increases with increasing growth temperatures for most hyperthermophilic archaea.^53^ GDGTs can undergo modifications beyond cyclization, including crosslinking and methylation, yielding structurally distinct GDGT lipid analogs.^54^ Enzymes implicated in the formation of GDGT-lipids are described below.

### 2.1 Tetraether synthase (Tes)

Unlike eukaryotic and bacterial membrane lipids, which are synthesized from straight-chain FA, archaeal lipids are prenols and are thus synthesized from isoprenoid building blocks to form branched carbon chains known as phytanyl. In some archaea, such as the thermophile *Methanocaldococcus jannaschii* (*M. jannaschii*), the phytanyl chains of a diether GDGT precursor, diether archaeal lipid, are tethered *via* a C–C bond to form a biphytanyl chain. This biphytanyl chain formation can occur either intermolecularly to form GDGT lipids or intramolecularly to form macrocyclic diether lipids (mAG) (Fig. 3). Before 2022, each step of archaeal membrane lipid biosynthesis was well established, except for the enzymes involved in the formation of the biphytanyl chains. This lack of clarity led to disagreements over whether the biphytanyl chains were formed *via* unsaturated intermediates or *via* a route involving completely saturated hydrocarbons. However, regardless of the biosynthetic route, the formation would demand two sequential C–H activations on the terminal sp^3^-hybridized (C(sp^3^)) carbons. The inertness of the substrate would require challenging radical chemistry and a means of stabilizing the initially formed radical while generating the second.

**Fig. 4:**
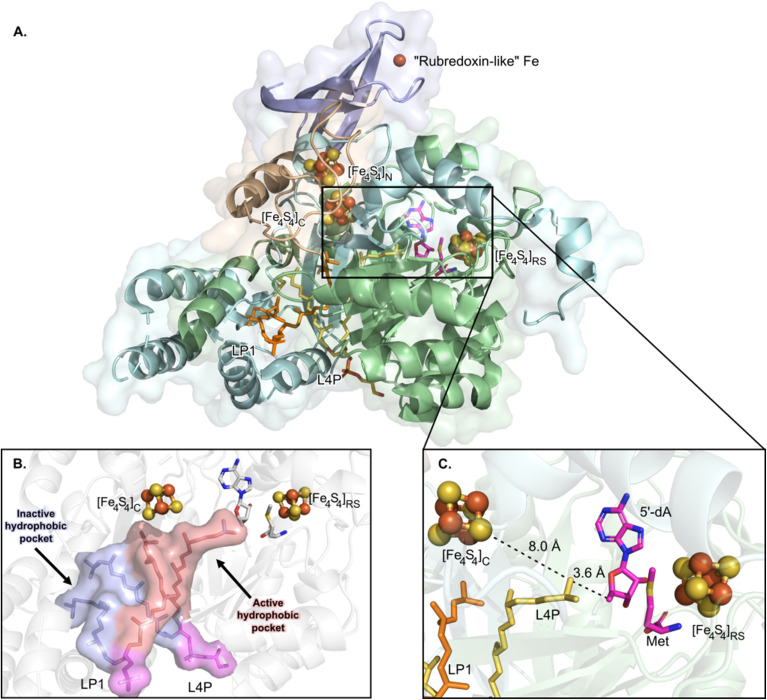
(A) Structure of Tes from *M. jannaschii* (PDB: 7TOL) comprised of four domains: the RS domain (blue), which houses the iron–sulfur cluster ([Fe_4_S_4_]_RS_) that participates in the reductive cleavage of SAM; a C-terminal domain (green), which contains a [Fe_4_S_4_]_C_; an N-terminal domain (orange), which contains a [Fe_4_S_4_]_N_ cluster; and a rubredoxin-like domain (purple), which contains a mononuclear Fe. (B) Hydrophobic pockets of Tes. (C) Distances between the terminal carbon of archaeal lipid substrate (L4P), 5′dAH, and [Fe_4_S_4_]_C_.

In 2022, Llyod *et al.* and Zeng *et al.* independently discovered the “missing link” responsible for biphytanyl chain formation in archaeal lipid biosynthesis.^30,55^ To uncover possible Tes candidates, Zeng *et al.* bioinformatically identified 18 genes annotated to encode RS proteins in *Sulfolobus acidocaldarius*. A homolog of one gene (Saci_0703) is located next to a ferredoxin known to activate geranylgeranyl reductase, an enzyme involved in lipid biosynthesis. Subsequent *in vivo* studies heterologously expressing a Saci_0703 homolog were performed in a *Methanococcus maripaludis* host, supporting the formation of the biphytanyl chains observed in macrocyclic diether lipid and GDGT. Simultaneously, Lloyd *et al.* identified and characterized Tes from *M. jannaschii* using X-ray crystallography (Fig. 4A) and *in vitro* activity assays. The crystal structure of Tes, with 5′-deoxyadenosine (5′-dAH) and methionine (PDB: 7TOL) bound, displayed density corresponding to two molecules of diether archaeal lipids with glycerol headgroups (archaetidylglycerol, AG) within two hydrophobic active-site pockets (Fig. 4B). This structure supports a lipid conformation conducive to GDGT formation where one phytanyl chain from each lipid substrate is present in an “active” pocket nearest to the [Fe_4_S_4_]_RS_ and [Fe_4_S_4_]_C_ clusters, while the other phytanyl chains reside in “inactive” pockets. The structure also displayed the terminal carbon of one of the phytanyl chains to be 3.6 Å from 5′-dAH, the product formed after the 5′-dA· abstracts a H·, suggesting that H· abstraction occurs at the very end of the phytanyl chain (Fig. 4C).

The Tes structure comprises four domains, each with a unique metallocenter: a rubredoxin-like domain, an N-terminal domain, an RS core domain, and a C-terminal domain. The rubredoxin-like domain contains an Fe^2+/3+^ ion, while the remaining domains each contain one [Fe_4_S_4_] cluster. Interestingly, each metallocenter has atypical ligand arrangements. Instead of four Cys ligands, as anticipated for a rubredoxin Fe and [Fe_4_S_4_] clusters, the rubredoxin-like iron and Fe/S cluster in the N-terminal domain ([Fe_4_S_4_]_N_) are each coordinated by 3Cys and 1His ligands, while the Fe/S cluster in the C-terminal domain ([Fe_4_S_4_]_C_) is coordinated by 3Cys and 1Met ligand. By contrast, the [Fe_4_S_4_]_RS_ is coordinated by 3Cys ligands, allowing for the unique iron to be coordinated upon SAM binding.

*In vitro* analysis of Tes activity with a chemically synthesized AG substrate showed a time-dependent formation of 5′-dAH, indicating radical chemistry from the reductive cleavage of SAM.^30^ The resulting lipids were also profiled and structurally characterized by high-resolution tandem mass spectrometry (MS/MS), revealing three distinct products: mAG, GDGT, and glycerol trialkyl glycerol tetraether (GTGT), a presumed intermediate species in forming GDGT. GDGT is produced from linking four C(sp^3^)–C(sp^3^) carbons to form two bonds, while GTGT is produced from linking two C(sp^3^)–C(sp^3^) carbons to form one bond. The formation of each C(sp^3^)–C(sp^3^) bond requires two H· abstractions mediated by two 5′dA·s; however, the enzyme's structure shows only a single SAM binding pocket. Therefore, after forming the first substrate radical, the reductive cleavage products of SAM must dissociate to allow a second molecule of SAM to bind, necessitating a way to stabilize the first substrate radical intermediate. Radical stabilization could occur through two proposed strategies: loss of a proton and an electron to generate an olefin intermediate or coupling of the radical to an auxiliary cluster. The active site contains a highly conserved Tyr residue that, in its basic form, could accept a proton en route to forming the olefin. In turn, the [Fe_4_S_4_]_C_ cluster could accept the electron. However, altering the Tyr to Phe or Leu did not significantly reduce the activity of the protein. By contrast, when the WT enzyme was incubated under turnover conditions but with limiting SAM, a novel peak was observed. This peak displayed an *m*/*z* consistent with the attachment of one sulfur atom on the AG substrate. This sulfur atom was presumed to derive from the [Fe_4_S_4_]_C_ cluster, suggesting that the substrate radical intermediate is stabilized by adding to a sulfur ion in the [Fe_4_S_4_]_C_ cluster to yield a substrate-cluster intermediate *via* a C–S bond. Upon quenching the reaction with acid, the [Fe_4_S_4_]_C_ cluster degrades; however, the C–S bond remains intact.

**Fig. 5:**
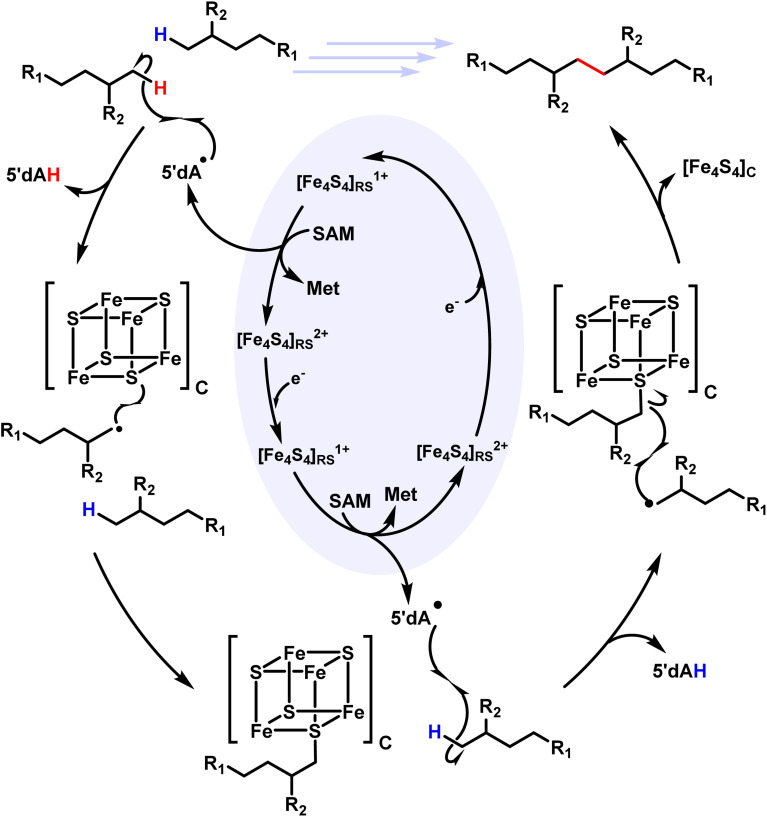
Proposed [Fe_4_S_4_] cluster-dependent radical coupling mechanism for Csp^3^–Csp^3^ bond formation observed in Tes,^30^ and postulated for Gms and Mss. (Aux = auxiliary [Fe_4_S_4_] cluster).

From these results, Lloyd and co-workers proposed the following mechanism for forming the biphytanyl chain in archaeal lipid biosynthesis. Upon reduction of the [Fe_4_S_4_]_RS_ cluster, SAM is cleaved, generating the 5′-dA·, which abstracts a H· from the terminal carbon of the phytanyl chain in the active hydrophobic pocket (Fig. 5). The resulting substrate radical then couples with a sulfur atom of the [Fe_4_S_4_]_C_ cluster, stabilizing the radical. After the loss of Met and 5′-dAH, a second SAM molecule binds and is reductively cleaved to generate a second 5′-dA·. This second 5′-dA· abstracts a H· from the second phytanyl chain, and the resulting substrate radical then attacks the carbon bonded to the sulfur of the [Fe_4_S_4_]_C_ cluster. This step results in C–C bond formation and liberation of the [Fe_4_S_4_]_C_ cluster. Based on this mechanism, generating the smaller macrocycle product, mAG, requires two SAM equivalents, while generating the GDGT-0 lipid requires four SAM equivalents, as two C–C bonds are formed (Fig. 5). The Tes reaction is also supported by *in vivo* studies of a Tes homolog from *Thermococcus kodakarensis*.^56^ Liman and co-workers observed that deletion of *T. kodakarensis* Tes resulted in decreased cell survivability at high temperatures and the loss of all GDGT lipids, again, emphasizing Tes's critical role in archaeal thermostability.^56^

### 2.2 Membrane-spanning lipid synthases (Mss)

Tes homologs have also been detected bioinformatically in some bacterial genomes. Shortly after the identification of Tes, Sahonero-Canavesi and co-workers identified two RS membrane-spanning lipid (MSL) synthases from *Thermotoga maritima* (WP_129545148.1) and *Thermotoga ethanolicus* (Tmari_0825).^52^ While mechanistic studies have yet to be performed, activity studies support that these enzymes carry out a similar reaction in bacterial monolayer lipids to form branched GDGT (Fig. 3A: brGDGT). The anaerobic expression of the *mss* genes from *T. ethanolicus* and *T. maritima* in *E. coli* led to the formation of two dicarboxylic acids (DA), absent in the control experiment. The *T. ethanolicus* Mss produced two predominant products, a C33-containing lipid with one cyclopropyl moiety and a C34 DA containing two cyclopropyl (*cy*) moieties. This result suggests a coupling of an *n*-C16:0 FA with a cy-C17:0 FA and the coupling of two *cy*-C17:0 FAs, respectively. Similarly, the expression of *T. maritima* Mss resulted in two predominant products: a C32 DA, from coupling an *n*-C16:0 FA with an *n*-C16:0 FA, and a C33 DA, by joining a *n*-C16:0 FA with a cy-C17:0 FA. Other DAs were also formed in lower relative abundances.

### 2.3 Glycerol monoalkyl glycerol tetraether synthase (Gms)

After GDGT-0 biosynthesis, (C)sp^3^–(C)sp^3^ crosslinking modifications of the biphytanyl chain forms glycerol monoalkyl glycerol tetraether (GMGT) lipids, also referred to as H-GDGT lipids (Fig. 3A: GMGT-0).^54,57^ Like GDGT, GMGT lipids are structurally diverse, including up to four cyclopentyl rings, and are found in ecological niches.^57^ While GMGT lipids are more prevalent in hyperthermophiles, their physiological role is poorly understood. A study in *Pyrococcus furiosus* shows that increased hyperthermophile growth-stress factors (acidic conditions, salinity, and elevated temperature) lead to increased GMGT lipid formation, implicating their role in membrane stability.^58^ While the exact location of the nascent carbon–carbon bond is unknown, it is proposed to occur between the C-20 and C-20′ methyl carbons, bridging the two alkyl chains of GDGT-0 to form GMGT-0.^59^

To identify enzymes catalyzing the formation of GMGT lipids, Garcia *et al.* performed a genomic search for RS homologs in three GMGT-producing organisms (*Thermococcus celers*, *Thermococcus guaymasensis*, and *P. furiosus*) that lacked homology with RS enzymes in *T. kodakarensis*, an organism that does not produce GMGT.^59^ This analysis yielded three putative GMGT synthases (Gms). To confirm their assignments, Garcia *et al.* inserted each candidate gene encoding the homologs (*Tc-gms*, *Tg-gms*, and *Pf*-gms) into a pTS549 plasmid, which was then used to transform *T. kodakarensis*. As expected, wild-type *T. kodakarensis* produced no appreciable GMGT lipids. However, upon heterologous expression of genes on the pTS549 plasmid, the GMGT lipid was the most abundant membrane-spanning lipid, comprising 73%, 70% and 59% of membrane-spanning lipids with *Tg*-Gms, *Tc*-Gms, and *Pf*-Gms, respectively.

Simultaneously, Li and co-workers conducted *in vivo* studies using a *Methanococcus maripaludis* strain containing the *tes* gene in which they heterologously expressed a Gms homolog from *Methanothermococcus okinawensis* (MoGms).^60^ Due to the presence of Tes, *M. maripaludis* can produce the GDGT-0 lipid substrate for MoGms. The authors observed the formation of GMGT-0, indicating that MoGms is responsible for the biosynthesis of GMGT lipids. Subsequent *in vitro* assays of MoGms in the presence of a lipid extract containing GDGT-0 demonstrated the formation of GMGT lipids, confirming the *in vivo* results. MoGms aligns well with Tes, which contains a SPASM domain. SPASM domains (named after the founding members of this RS subclass, which are involved in the maturation of subtilosin A, pyrroloquinoline quinone, anaerobic sulfatase, and mycofactocin) are C-terminal extensions of the canonical RS domain and allow binding of two auxiliary clusters.^33^ These results suggest that MoGms also contains two additional [Fe_4_S_4_] clusters. Li and coworkers aligned an AlphaFold-predicted structure of MoGms with the SPASM domain-containing RS enzyme SuiB to identify groups of cysteines that likely ligate each cluster ([Fe_4_S_4_]_RS_, [Fe_4_S_4_]_AuxI_, [Fe_4_S_4_]_AuxII_). Cysteines coordinating each cluster were altered to strategically remove specific metallocenters. These variants were then studied by electron paramagnetic resonance (EPR) spectroscopy upon reduction of the clusters with dithionite in the presence or absence of SAM. The spectrum of wild-type (WT) MoGms was largely axial; however, the addition of SAM significantly altered the spectrum, revealing at least two distinct cluster species. When both [Fe_4_S_4_]_AuxI_ and [Fe_4_S_4_]_AuxII_ clusters were removed, the EPR spectrum resembled the WT component that had bound SAM. When the [Fe_4_S_4_]_RS_ cluster and either [Fe_4_S_4_]_AuxI_ or [Fe_4_S_4_]_AuxII_ cluster were removed, the resulting spectra were nearly identical, suggesting that [Fe_4_S_4_]_AuxI_ and [Fe_4_S_4_]_AuxII_ exhibit similar EPR parameters. No spin quantification was reported in this work. The observations by Li *et al.* are consistent with the presence of three [Fe_4_S_4_] clusters as observed in Tes. Moreover, Li *et al.* proposed that the mechanism of C(sp^3^)–C(sp^3^) bond formation by Gms is likely similar to that of Tes. Therefore, Gms could utilize one of the auxiliary Fe/S clusters in this mechanism to stabilize the first substrate radical upon the second activation, yielding a substrate-cluster intermediate during catalysis. One interesting observation, however, Gms does not contain a rubredoxin domain.

### 2.4 GDGT ring synthase (GrsA and GrsB)

GDGT lipids can contain up to eight cyclopentane rings, which are constructed by forming carbon–carbon bonds between a methyl group and a methylene unit four carbons away. The increased presence of cyclopentane rings results in heightened membrane rigidity by enhancing packing density.^61^ Therefore, thermal stability is enhanced, and the membrane exhibits reduced ion permeability. The number of rings added to the GDGT core increases with an increase in growth temperature, allowing the utilization of cyclized GDGT lipids as paleothermometer biomarkers (*i.e.*, indicators of ancient environmental temperatures).^62,63^ Due to the inert characteristics of the GDGT, Zeng *et al.* hypothesized that cyclization would occur by a radical mechanism, often observed in members of the RS superfamily.^64^ Motivated by this hypothesis, they searched the *S. acidocaldarius* genome for members of the RS superfamily, identifying three putative RS enzymes (GeneIDs: *saci_0240*, *saci_1585*, and *saci_1785*) from organisms known to produce cyclopentane-containing GDGT lipids.

Gene deletions of the identified RS enzymes were performed in *S. acidocaldarius*. The deletion of *saci_1785* did not affect the formation of cyclopentane ring-containing tetraether lipids. By contrast, individual deletions of *saci_0240* and *saci_1585* significantly reduced cyclopentane ring formation. Deleting *saci_0240* and *saci_1585* together resulted in the complete loss of cyclopentane ring formation. These results suggested that *saci_1585* and *saci_0240* encode GDGT ring synthases (Grs), designated GrsA and GrsB, respectively. GDGT lipids can harbor up to eight cyclopentane rings, with four on the C7 and four on the C3 positions based on the numbering of the precursor diether lipids. GrsA catalyzes the formation of 1 to 4 cyclopentane rings on C7, while GrsB subsequently catalyzes the formation of an additional 1 to 4 cyclopentane rings on C3, yielding a total of 5–8 cyclopentane rings on GDGT (Fig. 3A: GDGT-8). Furthermore, GrsB exhibits very poor activity on GDGT-0, indicating that GDGT-0 is not the preferred substrate for GrsB.

**Fig. 6:**
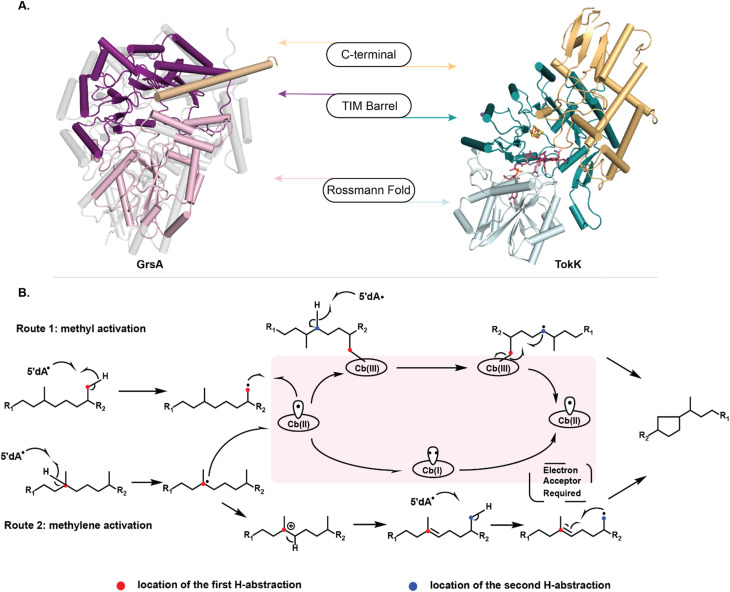
(A) (left) Alignment of AF model of GrsA (GeneID: saci_1585; pink – Rossmann fold; purple – TIM barrel; light orange – C-terminal helix) and GrsB (GeneID: saci_0240, transparent grey). (right) Overall structure of TokK (PDB: 7KDX) with both metallocofactors bound. Light blue – Rossmann fold; teal – TIM barrel; gold – C-terminal domain; raspberry – OHCbl; yellow – sulfur; orange – iron. (B) Proposed mechanisms of Grs proteins. Route 1: reaction is initiated at the methyl group of the biphytanyl chain, substrate radical is stabilized by forming a covalent intermediate with the Cbl cofactor, the formation of second substrate radical will initiate homolytic cleavage of the covalent Co–C intermediate bond; Route 2: reaction is initiated on the biphytanyl chain, the substrate radical donates the free electron to Cb(II) to form an olefin intermediate for stabilization, and the second substrate radical will initiate the cyclization.

Sequence similarity networks published on https://radicalSAM.org place GrsA and GrsB in Megacluster 2–1, which contains RS enzymes harboring a B12-binding domain.^19^ Moreover, the AF models of GrsA and GrsB from *S. acidocaldarius* contain an N-terminal Rossmann-fold-like domain, often found in B12-binding RS enzymes, supporting the bioinformatics assignment. The structures also show partial triosephosphate isomerase barrels containing the canonical CX_3_CX_2_C RS motif for binding an [Fe_4_S_4_] cluster, and a C-terminal α-helix, predicted to be involved in substrate binding (Fig. 6A).

Cobalamin (B_12_) consists of a cobalt ion (Co) embedded in a corrin ring.^65,66^ The cobalt ion can exist in oxidation states of +1, +2, and +3, giving rise to cob(I)alamin, cob(II)alamin, and cob(III)alamin, respectively. Cob(I)alamin, considered the strongest nucleophile in nature, has a lone pair of electrons in the d_*z*^2^_ orbital of the Co ion,^67^ while cob(II)alamin has one unpaired electron. The ability to form cob(II)alamin is central to the radical reactions in which the cofactor participates. Cob(III)alamin is often ligated by methyl, 5′-deoxyadenosyl, or hydroxyl moieties, affording methylcobalamin (MeCbl), adenosylcobalamin (AdoCbl), and hydroxo(aqua)cobalamin (OHCbl).^68^ B12-dependent RS methylases, except for TsrM,^69,70^ take advantage of all three oxidation states of cobalamin. B12 is also used by some RS enzymes to catalyze other reactions; however, the cobalamin oxidation states involved in these mechanisms are unknown.

Although GrsA and GrsB catalyze cyclopentane ring formation at different positions of GDGT, their AF models share structural similarity (Cα alignment RMSD = 1.264 Å), and the enzymes are predicted to operate by similar catalytic mechanisms. To form the ring, two inert C–H bonds must be activated by H· abstraction, requiring the formation of two 5′dA· per round of catalysis (Fig. 6B). Like Tes from *M. jannaschii*, GrsA and GrsB contain a single SAM binding pocket in which it associates with the [Fe_4_S_4_]_RS_ cluster to produce the 5′-dA·. Therefore, H· abstraction occurs in two distinct steps, necessitating that the first substrate radical be stabilized before generating the second substrate radical. Unlike Tes, GrsA and GrsB are predicted to lack an auxiliary Fe/S cluster to which the first substrate radical can associate for stabilization, making the Cbl cofactor an attractive candidate for this role.

Here, we propose two ways in which the Cbl cofactor in Grs proteins could be used in catalysis. In the first mechanism, cob(II)alamin recombines with the nascent substrate radical, generated by abstracting an H· from a target methyl moiety. This step stabilizes the substrate radical *via* a weak cobalt–carbon bond, forming an alkyl-cob(III)alamin. Upon generating the second substrate radical through H· abstraction by a second 5′dA·, the substrate radical attacks the Co-bonded carbon of the alkyl-cob(III)alamin intermediate, inducing its homolytic cleavage to afford the cyclized product and cob(II)alamin. A similar mechanism can be drawn with initial H· abstraction occurring at the methylene carbon; however, linking a primary carbon would be more sterically favorable. In the second proposed mechanism, cob(II)alamin acts as an electron acceptor. Upon generating the first substrate radical intermediate, the unpaired electron is transferred to cob(II)alamin to afford cob(I)alamin and a substrate carbocation, which is deprotonated to give an olefin. A second substrate radical, generated through a H· abstraction by a second 5′-dA·, attacks the olefin, generating the cyclized product containing an unpaired electron. Catalysis is completed upon the donation of an electron from cob(I)alamin and a proton.

### 2.5 GMGT methylase (Gmm)

**Fig. 7:**
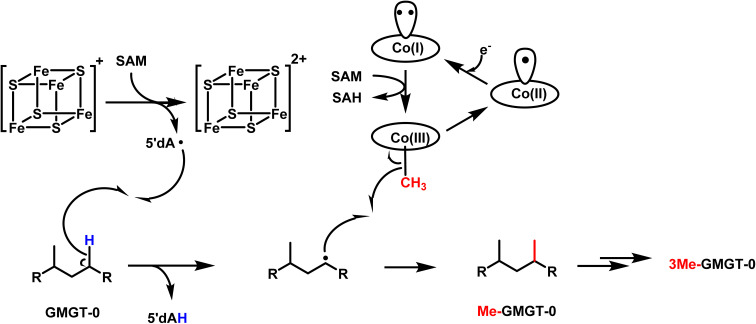
Proposed mechanism of Gmm-catalyzed iterative methylation on GMGT-0. This reaction requires two SAM molecules to react with both the [Fe_4_S_4_] cluster (for reductive cleavage) and the Cbl (for methyl donation).

Garcia and co-workers further inspected the genomic neighborhood of the *gms* gene in *T. guaymasensis* and uncovered neighboring genes encoding a Grs protein and a B_12_-binding RS methylase (*gmm*).^59^ The *gmm* gene encodes for GMGT methylase (Gmm), whose presence, when coexpressed with Gms, results in mono-, di, and tri-methylated GMGTs (Fig. 3: 3Me-GMGT-o). As a putative Cbl-dependent RS methylase (RSM), Gmm is surmised to operate *via* the mechanism shown in Fig. 7. In the first step of catalysis, Gmm will use SAM to methylate cob(I)alamin, yielding MeCbl and S-adenosylhomocysteine (SAH). After dissociation of SAH, a second SAM molecule will bind and be reductively cleaved to form a 5′dA·. The 5′dA· will then abstract a H· from the substrate alkyl chain to afford a substrate radical, which attacks the methyl moiety of MeCbl, inducing a homolytic cleavage of the Co–C bond and yielding the methylated product. The resulting cob(II)alamin is presumed to be reduced to cob(I)alamin by a reductant, preparing the enzyme for a subsequent round of catalysis.

While the exact locations of these methylations is unknown, MS/MS analysis suggests they occur on the alkyl chain of the GMGT and not on the glycerol backbone. These studies also suggest that Gmm prefers to methylate GMGTs over unbridged GDGTs, and/or must interact with Gms to function properly. As a means to understand whether Gmm activity depends on its association with Gms, an inactive variant of Gms lacking the [Fe_4_S_4_]_RS_ cluster was generated and co-expressed with WT Gmm. This experiment allowed Gmm to contact Gms without competing for the GDGT substrate. However, limited methylation activity was observed, indicating that GDGT-0 is not the preferred substrate of Gmm. Instead, Gmm prefers to methylate the Gms product, GMGT.

### 2.6 Calditol synthase (Cds)

**Fig. 8:**
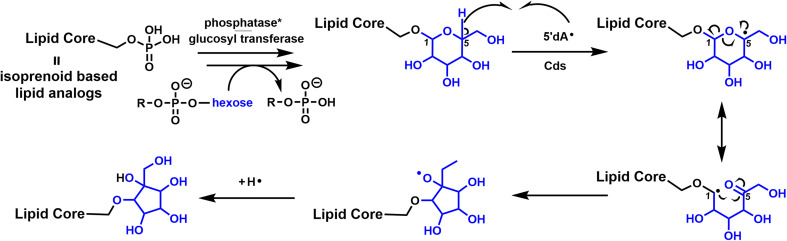
Proposed mechanisms of Cds-catalyzed ring contraction based on the previous studies on QueE.^31^ *The canonical glucosyl transferase mechanism necessitates that the lipid substrate be a primary alcohol, alluding to the biosynthetic involvement of an unknown phosphatase.^76^

RS enzymes are also involved in functionalizing and installing diverse head groups on lipids. Archaea possess a variety of head groups, including ethanolamine, serine, and myo-inositol linked to the glycerol backbone through a phosphate, or linked to the glycerol backbone through a sugar.^71,72^ Of the head groups, calditol is among the least studied. Calditol (Fig. 8) is a cyclopentapolyol polar head group that, when ether-linked directly to one or both glycerol backbones of a GDGT lipid, forms glycerol dialkyl calditol tetraether (GDCT) lipids. GDCT was first discovered in the thermoacidophile *S*. *acidocaldarius*, comprising the majority of the polar membrane lipids.^73,74^ However, before the work of Zeng and co-workers, it was unknown what physiological role this head group plays and which enzymes install it.^75^

Many studies suggest that calditol is derived from glucose, most likely through an ‘inositol-like’ pathway. This pathway involves an aldol-type condensation between C-1 and C-5 of glucose. However, an alternative radical-based mechanism exists where calditol is formed *via* a ring contraction strategy like that observed in the RS enzyme CDG synthase (QueE) during the biosynthesis of 7-carboxy-7-deazaguanine (CDG).^31^ With this in mind, Zeng *et al.* performed a genomic search of the *S. acidocaldarius* genome for members within PF04055, representing the RS superfamily within the protein family database (PFAM). From this analysis, they identified 18 hits, which they narrowed to three by excluding hits that did not have homologs with greater than 30% identity in other calditol-producing archaea. Independent genomic knockouts for the two punitive RS enzymes, Saci_0343 and Saci_1489, were generated in *S. acidocaldarius*. Following the growth of these mutants, the cells were subjected to acid hydrolysis, and the lipids were analyzed by LC-MS. Calditol head groups are uniquely resistant to acid hydrolysis due to the presence of the ether linkage, while other prevalent head groups (*e.g.*, phosphorylated and hexose) are not. WT and *Δsaci_0343* displayed MS traces exhibiting both calditol-GDGT lipids and GDGT core lipids. At the same time, *Δsaci_1489* contained only GDGT core lipids, indicating that the gene product of *saci_1489 (cds)* is a RS enzyme, known as calditol synthase (Cds), responsible for producing calditol. Therefore, the proposed mechanism for the ring contraction observed during the conversion of glucose to calditol is initiated by abstracting an H· from C-5 of the glucose ring to generate a substrate radical (Fig. 8). The presence of the C-5 radical induces ring-opening to generate a C-5 carbonyl with the unpaired electron relocated to the C-1 carbon. Subsequently, the C-1 radical couples with C-5 to generate an alkoxy radical, which abstracts a H· to produce the ring-contracted calditol product. Although more experiments are needed to validate this mechanism, the physiological role of calditol is supported by *S. acidocaldarius* (WT and Δ*cds*) growth studies under extreme acidic conditions. Not only did these results show that the inability to produce calditol (ΔCds) hindered growth at low pH values (pH 1.6), but bioinformatic analysis also indicated that Cds homologs are present in organisms primarily isolated from acidic environments. These studies suggest that calditol headgroups enhance membrane stability at extremely low pH values.

## 3. Hopanoid lipids

**Fig. 9:**
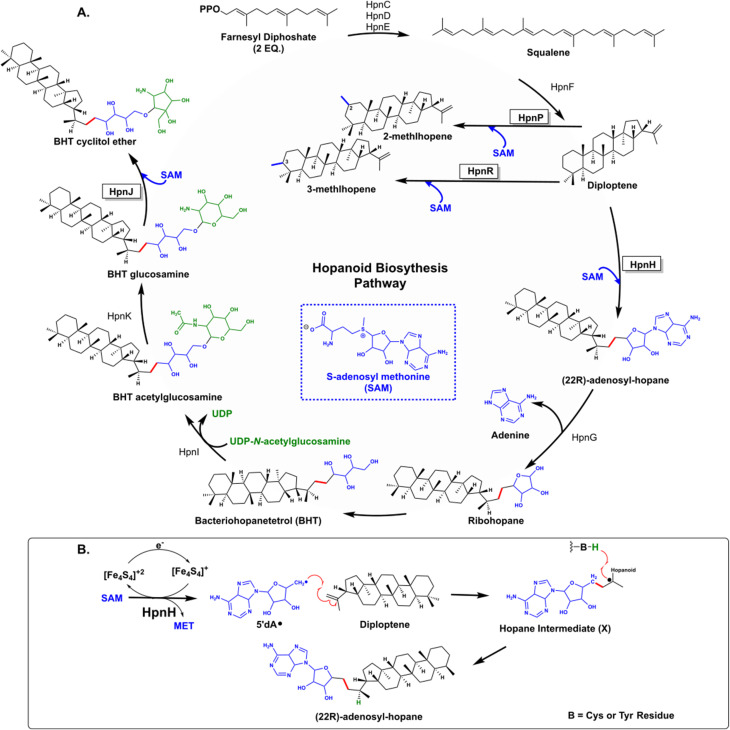
(A) Proposed hopanoid biosynthesis pathway in Burkholderia cenocepacia (https://doi.org/10.1111/1462-2920.12509). HpnD, HpnC, and HpnE catalyze squalene production from two molecules of farnesyl diphosphate. Squalene is then cyclized by squalene-hopene cyclase (SHC), HpnF. An adenosyl sidechain is attached *via* RS HpnH and truncated to ribosylhopane by putative nucleoside phosphorylase, HpnG. Ribosylhopane is predicted to be nonenzymatically transformed into BHT, which is functionalized with an *N*-acetyl-d-glucosamine sidechain extension by HpnI. HpnK catalyzes the deacetylation of the side for subsequent production of BHT cyclitol ether by punitive RS HpnJ. Boxed enzymes indicate RS enzyme. The red bond indicates Csp^3^–Csp^2^ bond formation. Bold blue bond indicates site-specific methylation on hopanoid ring “A”. (B) The proposed mechanism of C–C formation catalyzed by HpnH.^78^

Hopanoids are bacterial sterol-like lipids (cyclic triterpenoids) comprising polycyclic hydrocarbons with five rings: A–E (Fig. 1A). Like eukaryotic sterol lipids, hopanoids are widely used as biomarkers to monitor microorganism fitness over time. The genes encoded in the *shc* (squalene-hopene cyclases (SHC)) gene cluster are part of a highly conserved *hpn* (hopanoid biosynthesis) operon. In bacteria, they typically consist of four highly conserved genes: *hpnC, hpnD, hpnE, and hpnF* (Fig. 9A).^77^ These SHCs are necessary to generate squalene, the primary hopanoid precursor, and cyclize it to the sterol core. Squalene is first generated using two farnesyl diphosphates by the bacterial squalene synthase system, consisting of HpnD, HpnC, and HpnE.^77,78^ HpnD condenses two molecules of farnesyl diphosphate to presqualene diphosphate. Hydrolytic rearrangement catalyzed by HpnC converts presqualene diphosphate to hydroxysqualene, which is then dehydrated and reduced to squalene by HpnE. A notable difference in eukaryotes is that a single SHC enzyme typically catalyzes the three steps from farnesyl diphosphate to squalene, indicating that hopanoid production is a highly regulated pathway in bacteria. After squalene production, squalene-hopene cyclase, HpnF, cyclizes squalene to a pentacyclic triterpenic olefin diploptene (DPT), completing the formation of the hopanoid core.

Downstream from the *shc* genes lie genes encoding enzymes for hopanoid structural modifications.^37^ As hopanoid structures vary in methylation at the C-2 or C-3 position, level of unsaturation, and/or sidechain functionalization, these enzymes often require radical chemistry to activate inert C–H bonds, as is typically observed in RS enzymes.

### 3.1 HpnH

Hopanoid side chains are hydrophobic and hydrophilic groups that attach to the hopanoid lipid core. Variations in these side chains increase the diversity and function of hopanoids. The most common hopanoid modification is the addition of hydrocarbon side chains to the olefin of DPT.^79^ This reaction is initiated by the RS enzyme HpnH, which is highly conserved across almost all hopanoid-producing bacteria. Hopanoids made by HpnH are known as the extended (C35) hopanoids, and they are formed by adding C30 hopanoids to C-5 of ribose moieties. HpnH catalyzes the first step of this extension *via* C–C bond formation by adding an adenosyl moiety to DPT in the form of adenosylhopane.^79^*hpnH* knockouts in *Streptomyces*,^80^*Burkholderia*,^37^*Rhodopseudomonas*,^81^ and *Methylobacterium*^79^ resulted in observable DPT. The corresponding absence of the C35 hopanoid confirmed HpnH's function.

It was assumed that HpnH followed a mechanism like that of aminofutalosine synthase (MqnE). MqnE is an RS enzyme that catalyzes the addition of a 5′-dA· to the terminal double bond of 3-[(1-carboxyvinyl)oxy]benzoic acid, which is then rearranged and decarboxylated to form aminofutalosine in *Streptomyces* menaquinone biosynthesis.^82^ Sato and co-workers provided support for this mechanism *via* deuterium-exchange studies that indicated that HpnH catalyzes stereoselective C–C bond formation to afford (22R)-adenosylhopane.^78^ In their proposed mechanism, SAM is reductively cleaved to form the 5′-dA·, which adds to the DPT terminal olefin at C22–C29. This step generates a C22 radical intermediate they designated “X.” They then proposed that X abstracts a H· from a residue in the active site, yielding adenosylhopane. Their studies further suggested that this residue can exchange protons with water (Fig. 9).

Further modifications are necessary to form the matured C35 hopanoid. After HpnH catalyzes the addition of the adenosyl moiety, HpnG, a purine nucleoside phosphorylase (PNP) homolog, cleaves adenine from adenosylhopane. This step was confirmed by *hpnG* knockouts, which result in the accumulation of adenosylhopane. PNP nucleoside cleavage requires a phosphate addition to the ribose of adenosylhopane;^83^ however, the theoretical product, phosphoribohopane, has yet to be observed experimentally for HpnG.

### 3.2 HpnJ

While the biosynthetic steps downstream of adenosylhopane differ among various organisms, the following steps of hopanoid synthesis in *Methylobacterium* involve opening the ribose ring. Subsequently, the majority of the resultant hopanoid is converted into bacteriohopanetetrol (BHT) cyclitol ether. In *Burkholderia cenocepacia,* BHT is also found as BHT cyclitol ether and BHT glucosamine functionalized hopanoid forms. Deletion of the *hpnJ* gene in *B. cenocepacia* resulted in undetectable BHT cyclitol ether, while the production of the other extended BHT hopanoids was maintained.^37^ Welander *et al.* hypothesized that HpnJ catalyzes the ring-contraction necessary to produce BHT cyclitol ether from BHT glucosamine. While the exact mechanism of the ring-contraction reaction in HpnJ has yet to be explored, as mentioned above, the RS enzyme QueE from *Bacillus subtilis* performs a ring contraction similar to that necessary for the conversion of BHT glucosamine to BHT cyclitol ether, and that proposed for *Cds* from *S. acidocaldarius* (Fig. 8).^31^

### 3.3 HpnP and HpnR

The late stationary survival of *Methylococcus capsulatus* is directly correlated to the production of C-3 methylated hopanoids,^84^ while the production of C-2 methylated hopanoids is important for surviving stress from nitrification in nitrite-oxidizing bacteria.^85^ HpnP and HpnR have been identified as B_12_-dependent RSMs responsible for producing 2- and 3-methylhopanoids, respectively.^84–86^ Zundel and Rohmer's 1985 bacterial feeding studies with labeled methionine ([*methyl*-^3^H,^14^C]-methionine, and [*methyl*-^2^H_3_]-methionine) identified SAM as the potential methyl donor for both 2- and 3-methylhopanoids, consistent with B_12_-dependent RSM mechanisms (Fig. 7).^87^ We propose that HpnP and HpnR perform a canonical B_12_-dependent RS methylation reaction as previously described for Gmm. However, detailed mechanistic studies have yet to be performed.

## 4. Jaw5

Jawsamycin (formerly known as FR-900848) is a potent antifungal agent that suppresses the growth of pathogenic fungi by targeting the fungal UDP-GlcNAc phosphatidylinositol transfer complex.^88^ Biosynthesized in *Streptoverticillium ferven HP-891*, jawsamycin's structure consists of a 5′-amino-5′-deoxy-5,6-dihydrouridine headgroup connected to a polycyclopropane-rich fatty acid. Feeding studies with [*methyl*-^13^C] methionine support that the methylene groups of the cyclopropane rings are sourced from methionine building blocks (*i.e.*, SAM), while the fatty acid backbone derives from the polyketide biosynthetic pathway.^89–91^ Hiratsuka *et al.* identified the jawsamycin biosynthetic gene cluster, consisting of nine open reading frames (*jaw1*–*jaw9*).^92^ Heterologous expression of the *jaw* genes in *Streptomyces lividans TK23* demonstrated that three enzymes, including an iterative type-I PKS (Jaw4), a *trans*-acting ketoreductase (Jaw6), and an RS enzyme containing a HemN-like domain (Jaw5), participate in the construction of the polyketide chains.^90,93^ HemN-like domains typically bind two SAM molecules simultaneously, where one is coordinated to the [Fe_4_S_4_]_RS_ by its carboxylate oxygen and amino nitrogen, while the other is peripheral to the [Fe_4_S_4_]–SAM complex in a second SAM binding site.^21,94^ The limited literature suggests that Jaw5 is uniquely responsible for the cyclopropane modification, and that this cyclopropanation occurs stepwise, directly after dehydration, rather than after a fully extended polyene is produced. This stepwise cyclopropanation is supported by additional feeding studies with deuterated synthetic diketide precursors, which led to the production of labelled product. Additionally, a Δ*jaw5* strain showed no cyclized product and no detectable polyunsaturated products.^92,95^

**Fig. 10:**
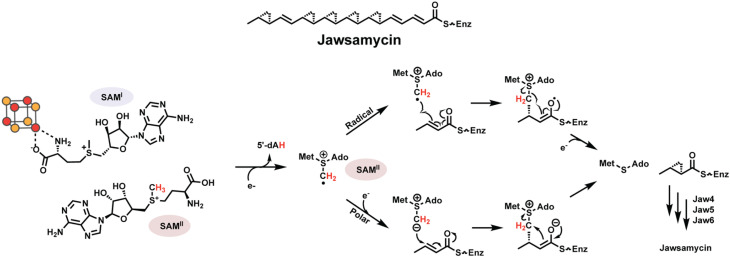
The proposed cyclopropanation mechanism performed by Jaw5 during the biosynthesis of jawsamycin.^28^

The Booker lab previously proposed two possible cyclization mechanisms: polar-based and radical-based.^28^ Consistent with Class C RS enzymes, both mechanisms would require the generation of a SAM methylene radical by a 5′-dA· formed from the cleavage of one SAM molecule, which would then abstract an H· from the methyl group of another SAM molecule.^21^ In the polar-based mechanism, an ylide is formed when the SAM methylene radical accepts an electron, which then attacks the substrate α/β-unsaturated carbonyl to generate a substrate carbanion. This carbanion attacks the bridging methylene carbon of SAM, yielding the cyclopropane motif and SAH (Fig. 10). The polar mechanism is akin to that of cyclopropane fatty acid synthases, which catalyzes the conversion of unsaturated fatty acids to cyclopropane fatty acids.^96^ In the radical-based mechanism, the SAM methylene radical adds to the α/β unsaturated carbonyl of the substrate, yielding a substrate radical. The substrate radical then attacks the bridging methylene carbon of SAM, yielding the cyclopropane substituent and SAH.^21^ Jin *et al.*, performed mechanistic studies involving mutagenesis, NMR, and MS/MS with a Jaw5 homolog, C10P, which provided support for the radical-based mechanism.^97^

## 5. Ladderane biosynthesis

**Fig. 11:**
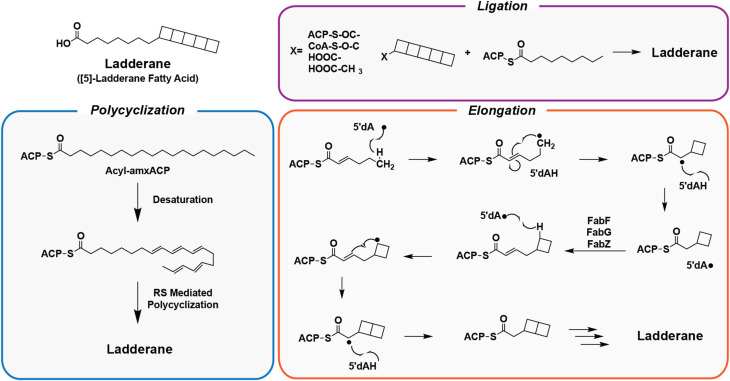
Three proposed mechanisms of RS enzyme mediate ladderane biosynthesis.^101^ RS-mediated polycyclization: The amxACP polyunsaturated intermediate is synthesized from a fatty acid using a desaturase to insert double bonds. The lipid is then folded into a ladderane, using an RS-dependent mechanism. (*blue*) RS-mediated ligation: the fatty acid and cyclized portion of ladderane are synthesized separately and ligated *via* RS-dependent C–C bond formation. (*purple*) RS enzyme-mediated elongation: the rings are formed as the acyl chain grows. (*orange*).

Anaerobic ammonium-oxidizing (anammox) bacteria are well-known for three distinguishable features: a unique intracytoplasmic membrane-bound organelle, known as the anammoxosome; the production of hydrazine (within the anammoxosome), an essential metabolite for the organism; and the biosynthesis of ladderane lipids, which contain multiple butane rings as cell membrane components (Fig. 11).^39,98,99^ Damsté, *et al.* first proposed that these three novel characteristics of anammox bacteria work together to support anammox catabolism based on their work on *Candidatus Kuenenia stuttgartiensis* (*K. stuttgartiensis*). In their work, the isolation of anammox bacterial membrane lipids first revealed the existence of ladderane lipids. NMR studies showed that ladderane lipids are C20 ketoacyl fatty acids that have 3 or 5 linearly continuous cyclobutane moieties attached to the C9 position. In contrast, the 3-cyclobutyl-inserted ladderane has an additional cyclohexane at the beginning of the ladder-shaped modification.^39,100^ Due to the highly condensed cyclic modifications, ladderane lipids present a higher density (1.5 kg dm^−3^) than regular cell membrane lipids (1.2 kg dm^−3^). Damsté *et al.* hypothesized that the anammoxosome membrane would require low ion permeability to retain the highly reactive and toxic hydrazine metabolite compartmentalized within this organelle. Due to increased membrane packing caused by ladderane lipids, these lipids were proposed to be the major component of the anammoxosome membrane, thereby decreasing permeability.^98^

As previously mentioned, low chemical reactivity is an innate property of hydrocarbon chains. Therefore, the formation of intramolecular cyclobutane rings during ladderane biosynthesis is proposed to be a radical-dependent process. Nature employs several strategies to initiate radical chemistry; however, many of these strategies necessitate oxygen. Due to anammox bacteria being strict anaerobes, RS chemistry is a likely candidate for ladderane biosynthesis. Although the enzyme(s) responsible for installing the cyclobutane rings remain unknown, the presence of uncharacterized RS enzymes within the genomic neighborhood of known proteins involved in fatty acid biosynthesis, such as ACPs, was used to identify the ladderane biosynthetic gene cluster. In several bioinformatics studies of the entire genome of *K. stuttgartiensis*, four operons encoding fatty acid biosynthesis proteins were identified, three of which contained putative RS enzymes.^39,99,101^ Moreover, recent studies on ladderane biosynthetic gene clusters provide better insight into the formation of these unique lipids. Results of these studies identified an anammox-specific acyl carrier protein (amxACP) to initiate the fatty acid biosynthesis, a FabZ homolog (amxFabZ) that catalyzes the dehydration of the hydroxyacyl-ACP precursor, a phytoene dehydrogenase (PDH)-like enzyme to form polyunsaturated hydrocarbon (PUHC) chains, two FabF-homologs (FabF2 and FabF) for chain elongation, and a B_12_-dependent RS enzyme proposed to catalyze the cyclobutane formation.^101^ It is believed that the RS enzyme plays a crucial role in catalyzing the formation of the contiguous cyclobutanes on ladderane lipids. The novelty of the ladderane structure has led to many proposed radical-dependent biosynthetic routes. Damsté *et al.* and Nouri *et al.* both hypothesize that the anammox could first generate a common saturated fatty acid chain. Saturated C_20_ will be dehydrogenated to PUHCs and then cyclized and rearranged to ladderanes (Fig. 11A). However, a single or spontaneous polycyclization route could be energetically unfavorable, potentially requiring an additional enzyme to stabilize the high-energy system. Currently, this pathway is unsupported by experimental data. By contrast, Rattray *et al.* proposed a pathway in which the linear part of the ladderane is constructed by canonical fatty acid synthesis, while the contiguous C20 cyclobutane is synthesized separately from the products of an unknown gene cluster. The two parts will then be fused together to yield ladderane using an FabF homolog (Fig. 11B). A third hypothesis suggests that cyclization could occur stepwise, akin to Jaw5, *via* a distinct RS-dependent mechanism. The reaction would start with an ACP-linked 2-butenic acid, which would then be activated at the terminal methyl group to initiate intramolecular cyclization. This will directly yield a cyclobutane on the ACP-linked intermediate that can be further elongated by FabF (Fig. 11C).

## 6. Conclusion

This review has summarized the current understanding of RS enzymes in lipid biogenesis and downstream functionalization. Because lipids are intrinsically unreactive, their modification often relies on radical chemistry. With the ability to readily activate C–H bonds, RS enzymes have played an integral physiological role in lipid biogenesis. Many questions remain about the exact mechanisms by which these enzymes catalyze their reactions. It is interesting to note that RS lipid modification is almost entirely associated with isoprenoid-based lipids. To our knowledge, ladderanes and the Jaw5 product are the only ketoacyl lipids proposed to be modified by RS enzymes. Discovering and characterizing more RS enzymes involved in complex lipid reactions will further expand the catalytic repertoire of the RS superfamily and aid in unveiling previously unknown lipogenesis pathways. Furthermore, understanding how RS enzymes modify lipids to increase stability could inform paleoenvironmental reconstruction and inspire pharmaceutical efforts for lipid-based drug delivery.

## 7. Conflicts of interest

There are no conflicts to declare.

## Data Availability

No new data was created during this study. Data sharing is not applicable to this article.
